# RETRACTION: Wound Healing and Postoperative Management in Paediatric Patients Following 27‐Gauge Transconjunctival Sutureless Vitrectomy for Vitreoretinal Conditions

**DOI:** 10.1111/iwj.70435

**Published:** 2025-03-26

## Abstract

**RETRACTION:** WenTaoD. , SanMeiL. , JieL. , and JieZ. , “Wound Healing and Postoperative Management in Paediatric Patients Following 27‐Gauge Transconjunctival Sutureless Vitrectomy for Vitreoretinal Conditions,” International Wound Journal 21, no. 4 (2024): e14611, 10.1111/iwj.14611.38156741 PMC10961879

The above article, published online on 29 December 2023, in Wiley Online Library (http://onlinelibrary.wiley.com/), has been retracted by agreement between the journal Editor in Chief, Professor Keith Harding; and John Wiley & Sons Ltd. Following an investigation by the publisher, all parties have concluded that this article was accepted solely on the basis of a compromised peer review process. The editors have therefore decided to retract the article. The authors did not respond to our notice regarding the retraction.

**RETRACTION:** D. WenTao , L. SanMei , L. Jie , and Z. Jie , “Wound Healing and Postoperative Management in Paediatric Patients Following 27‐Gauge Transconjunctival Sutureless Vitrectomy for Vitreoretinal Conditions,” International Wound Journal 21, no. 4 (2024): e14611, 10.1111/iwj.14611.38156741 PMC10961879

The above article, published online on 29 December 2023, in Wiley Online Library (http://onlinelibrary.wiley.com/), has been retracted by agreement between the journal Editor in Chief, Professor Keith Harding; and John Wiley & Sons Ltd. Following an investigation by the publisher, all parties have concluded that this article was accepted solely on the basis of a compromised peer review process. The editors have therefore decided to retract the article. The authors did not respond to our notice regarding the retraction.

